# Association between adverse childhood experiences and masculinity with well-being: moderating role of behavioural emotional regulation among men of three nations

**DOI:** 10.1038/s41598-025-99193-4

**Published:** 2025-04-28

**Authors:** P. Padma Sri Lekha, E. P. Abdul Azeez, Bhoomika N. Jadhav, Wafa Said Al-Maamari, Emad Farouk Saleh, A. P. Senthil Kumar

**Affiliations:** 1 School of Social Sciences and Languages, Vellore Institute of Technology, Vellore, 632014 India; 2 College of Arts and Social Sciences, Sultan Qaboos University, Muscat , Oman; 3 College of Social Science and Humanities, University of Jigjiga, Jigjiga, Ethiopia

**Keywords:** Masculinity, Adverse childhood experiences, Self-care, Self-compassion, Behavioural emotional regulation, Psychology, Health care

## Abstract

The psychosocial aspects of men’s health and well-being have gained attention in the literature in recent years. However, evidence from developing countries is limited. Therefore, the present study attempted to understand the determining role of Adverse Childhood Experiences (ACEs) and masculinity on well-being factors, namely self-care and self-compassion among men, along with the moderating role of behavioral emotional regulation (BER) between masculinity and self-care. We adopted a cross-sectional study design. The data were collected from three countries, which are patriarchal societies, namely Ethiopia, India, and Oman, with a total sample size of 823 men between 18 and 45 years. Self-reported measures of the key variables were administered among the participants. We performed descriptive statistical analyses and path analysis. The ACEs were positively associated with masculinity (b = 1.544; 99% CI = 1.227–1.853), while it reduced the likelihood of self-compassion. Further, the increase in masculinity increased self-care (b = 0.195; 99% CI = 0.097- 0.295). However, the use of negative BER strategies reduced the likelihood of involvement in self-care (b=-1.185; 95% CI= -2.280- − 0.125) and changed the direction between masculinity and self-care (b=-0.644; 95% CI = − 0.988- − 0.279) acting as a moderator (b = 0.027; 95% CI = 0.003–0.051). The results suggest the importance of BER in effectively promoting self-care among men. Future self-care programs and interventions in the three nations should consider training men in BER. BER-focused interventions can facilitate positive coping among men and further enhance self-care and self-compassion.

## Introduction

Enhancing the well-being of individuals has been an essential component of health promotion and a driving factor for various physical and mental health initiatives globally^1^. In this line, although the United Nations considers health and well-being for all ages a sustainable developmental goal, a significant gap exists in attaining these goals^2^. Further, this becomes essential with the increasing burden of non-communicable and mental health issues globally^3–5^. Maintaining good physical and psychological health throughout life is a challenge. However, involvement in self-care can promote health and well-being^6–8^. Self-care is a conscious activity that enhances one’s capability to improve health and reduces the risk of developing serious illnesses^9^. In addition, self-care includes habits, lifestyle choices, and everyday practices that reduce morbidity and mortality when done appropriately^10^.

Moreover, being compassionate with oneself is an essential factor that self-regulates one’s health-promoting behavior^11,12^and well-being^13^. A study among college students in the United States identified the protective role of self-compassion as it reduced the physical and psychological manifestation of stress^14^. Similarly, a longitudinal study among community dwellers identified compassion towards self and others as a significant predictor of physical and mental well-being across the lifespan^15^. It is evident from these studies that self-care and self-compassion are pivotal aspects of the health and well-being of individuals. However, these aspects are affected by various individualistic and social factors. One relevant factor associated with self-care and self-compassion is adverse childhood experiences (ACEs)^16,17^.

The traumatic events that individuals experience till 18 years of age are considered to be ACEs^18^. This covers a range of traumatic events^19^and is found to have a significant impact on individuals’ health practices and outcomes that increases the risk of involvement in unhealthy behaviors such as smoking, alcohol consumption, drug abuse, and sexual promiscuity during adolescence and adulthood^20–22^further paving the way for long-term health effects. In addition, ACEs also influence how things are perceived, creating a negative attitude toward the surroundings^23^, which in turn increases the risk of mental health issues as an adult^24–26^. Further, a study evidenced a negative relationship between ACEs and family health resources and emotional health processes in adulthood^27^. Interestingly, these experiences differed with gender, as a study among school students in Tunisia identified more exposure to physical abuse and all forms of ACEs among boys with a higher prevalence of internet addiction and lower scores on resilience compared to girls^28^.

In addition, gender differences existed in the association between ACEs and psychological distress. For boys, ACEs were associated with externalized and internalized psychological distress, while it was only related to externalized psychological distress among girls^29^. Along with this, gender norms played a key role in the health and well-being of individuals^30,31^, creating havoc for both men and women. The case of women concerning gender norms is widely studied. For men, conforming to traditional masculine norms can lead to compromised health, increasing involvement in risky behaviors^32,33^. In the same way, endorsing masculine honor and the masculine norm was associated with higher risk-taking behaviors among individuals^34,35^, sabotaging self-care. In addition, the masculine norm and men’s conformity reduced health literacy^36^and increased adverse mental health effects^37,38^. Although, in general, masculinity is related to reduced self-care and poor well-being among men, few recent studies have pointed to the positive dimensions of masculinity^39^. The evolving nature of masculinity is being discussed as positive masculinity because it promotes involvement in self-care and flexibility among men^40,41^.

However, traditional masculinity is still prevalent, as men perceive it to be valued by other men and consider it important in the ingroup context^42^. In addition, the endorsement of traditional masculine ideologies was positively associated with fear of emotions^43^, which increased aggression, domestic violence perpetration, and emotional suppression with reduced emotional competencies and low self-compassion among men^44^. This suggests it becomes essential for men to adopt positive behavioral emotional regulation (BER) strategies to enhance their self-care and well-being. Emotional suppression was particularly associated with depression among men than in women, though depressive symptoms are more prevalent among women^45^. On the other hand, men used more regulatory strategies when they perceived a situation as stressful and deserved emotional involvement^46^, which in turn might affect their self-care behaviors. The concept of BER is similar to coping strategies as both focus on managing and regulating emotions and behaviors during stressful situations^47^. Positive ways of regulation are considered to be adaptive and constructive which enhances overall well-being, while negative strategies are considered to be maladaptive and hold the potential to intensify stressful experiences^48^. These aspects are supported by the trauma theory where the experiences of trauma as ACEs could have a long-lasting negative impact^49^on the health, mental health and well-being by increasing the vulnerability of the individuals^50^ that the internalized masculine gender norms could further exacerbate.

Further, the intersectionality between ACEs and masculine norms was expected to be disadvantageous to men, harming their health and well-being. Although the discussed literature provides a foundation for selecting these variables and their association in isolation, to our knowledge, no earlier work has comprehensively considered the role of ACEs on masculinity and its role on BER, self-care and self-compassion. Men’s health and well-being have been majorly overlooked, with priority given to women and girls in global health organizations^51^. However, over recent years, there has been a greater focus on men’s health and mental health, especially in terms of self-care in Western countries. Still, a significant gap exists in understanding factors of men’s health and well-being (self-care and self-compassion) in developing nations. In this study, we considered India, Oman, and Ethiopia as these countries are essentially patriarchal societies with beliefs in traditional masculine norms reflected in these cultures. In addition, minuscule studies exist exploring men’s health and well-being in these countries. A study pointed to the role of country-level beliefs about gender on men’s health, suggesting that it is not just about men’s masculinity and their related beliefs but also the country-level belief about gender that plays a crucial role in men’s health^52^. Although Oman is considered economically secure, ACEs are prevalent, increasing the odds of adults’ involvement in risky behaviours^53^.

**Fig. 1:**
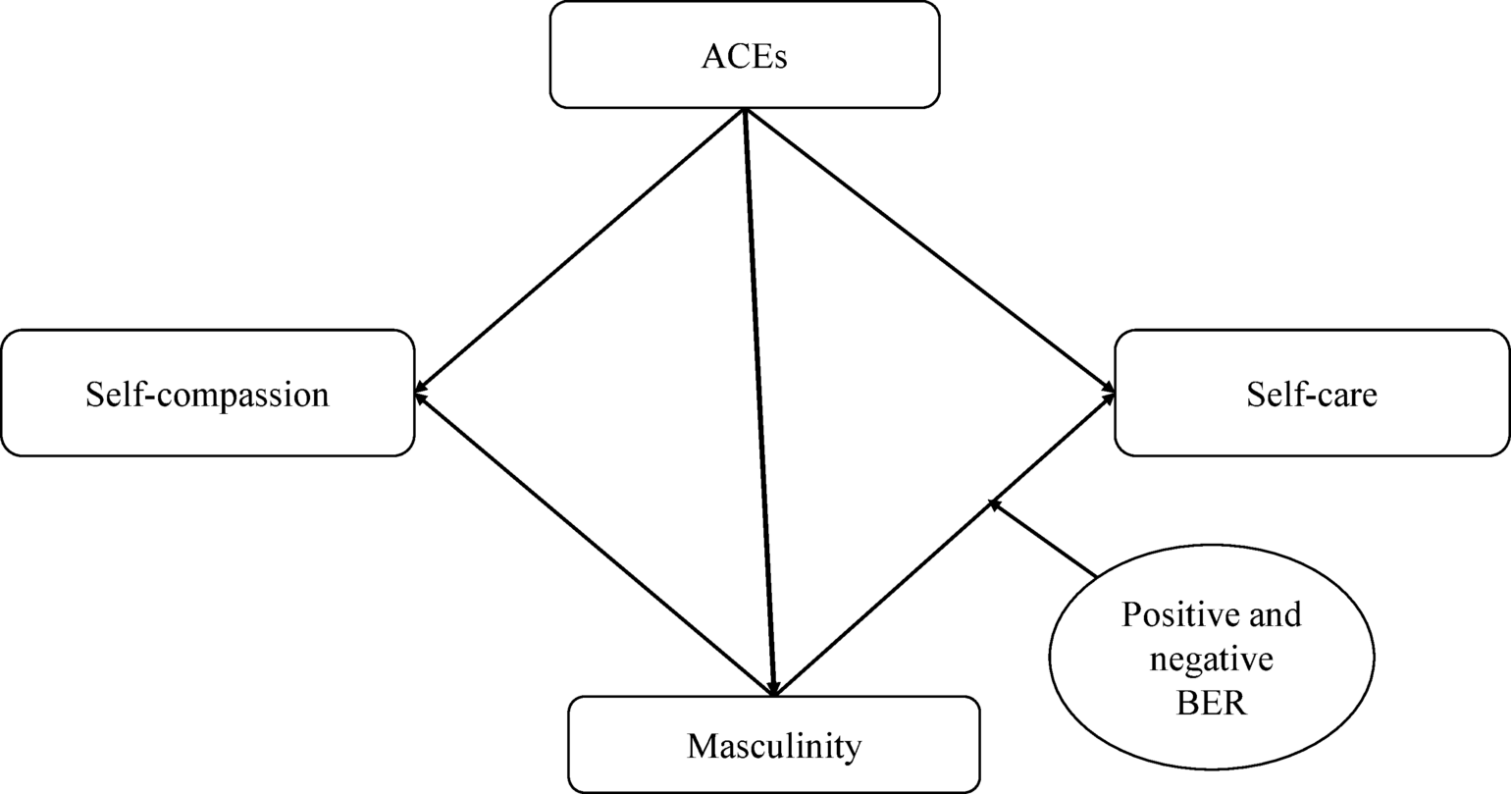
Conceptual framework of the study.

The countries considered for this study have strong gender norms prevalent in their cultures, impacting men and women. The traditional masculine norms prevailing in these societies influence men’s health and well-being. In addition, these countries evidence potential poor health behaviors among men in terms of alcohol consumption and tobacco use^54–57^that could be attributed to gender norms. This could increase the risk of non-communicable diseases (NCDs), justifying the need for understanding self-care among men. Furthermore, there exist poor healthcare policies for men^55^at the global front^58^, including these countries. With this background, the present study aimed to understand the determining role of ACEs and masculinity on self-care and self-compassion among men, along with the moderating role of behavioral emotional regulation (BER) between masculinity and self-care. The conceptual framework of the study is depicted in Fig. 1.

## Methods

### Study design and setting

We have adopted a cross-sectional study design involving selected localities from Ethiopia, India and Oman. The data used for this study was collected from community-dwelling men during February 2024 to August 2024.

### Participants and procedures

The participants were recruited from communities. In India, we have considered four communities (2 rural and 2 urban), while participants from Ethiopia (2 rural and 1 urban) and Oman (1 rural and 2 urban) were recruited from three communities. The communities were chosen randomly from a selected district in these three countries. The population of these communities varied from country to country, and it was between 3,000 and 5,000 approximately. We recruited the participants using systematic sampling techniques, where the potential participants were approached through the households in these communities in fixed sampling intervals. The selection of the samples was based on inclusion and exclusion criteria. First, men aged between 18 and 45 reside in the target area. Second, participants who can respond to the survey in English. Third, participants with severe physical and mental health conditions were excluded. The participants of the present study were 823 men (India = 321, Oman = 214, and Ethiopia = 288), (Mean age = 29; SD = 7.79).

After identifying the potential household and participant, the research team briefed the study’s aims and requested that the questionnaire be filled out electronically or by paper-and-pen method. The majority of the participants filled the questionnaire electronically (94%). The survey form included a tentative title of the present study, a brief description, participant informed consent, demographic details, and the measurements of adverse childhood experiences, masculinity contingency, behavioral emotional regulation, self-compassion, and self-care, all in English.

### Measures used

We considered self-care and self-compassion as factors of well-being, as reflected in the works of Martínez et al.^7^ and Cowand et al.^14^.

#### Outcome measures

*Self-Care inventory (SCI)*: This 20-item SCI measures self-care in the general adult population^59^. Participants could rate the items using a five-point Likert scale *(1 = never or not likely; 5 = always or very likely)* on all three subordinate scales- self-care maintenance, self-care monitoring, and self-care management. The total score ranges from 20 to 100. With a high score on this inventory indicating high self-care behaviors, the reliability coefficients of the above sub-scales were 0.85, 0.88, and 0.88, respectively. The full-scale indicated excellent reliability for this sample, with Cronbach’s alpha of 0.93.

*Self-Compassion scale (Short-form)*: Self-compassion measured by this scale assesses the capacity of individuals to experience one’s feelings of suffering with a sense of concern, warmth, and connection. A total of 12 items, covering self-kindness, self-judgment, common humanity, isolation, mindfulness, and over-identification with a five-point Likert response (*1 = almost never; 5 = almost always*), provide a score ranging from 12 to 60. A high score captures higher self-compassion. The test-retest reliability reported in the original study was.71^60^. Further, for this sample, the self-compassion scale indicated an excellent reliability (Cronbach α = 0.87).

#### Predictors

*Adverse childhood experiences questionnaire (ACE-Q)*: The 10-item ACE-Q^61^measured the adversities experienced during childhood, including abuse, neglect, and household-related challenges. Every item endorsed by the participant was awarded one point. An overall score on this questionnaire ranged from 0 to 10, with a high score indicating higher ACEs. The Cronbach’s alpha was 0.88^62^. In the present study, Cronbach’s alpha of ACE-Q was 0.90.

*Masculinity Contingency Scale (MCS)*: The MCS is a 10-item scale measuring men’s self-worth as derived from their sense of masculinity^63^. The scale has two categories: ‘Boost’ (confirming masculinity boosts men’s self-worth) and ‘Threat’ (defining their motivation to defend masculinity as a result of self-worth being threatened due to a lack of it) while also allowing them to obtain a total score on the scale. The measure has a seven-point Likert response pattern where 1 indicates strongly disagree while 7 means strongly agree. The maximum overall score obtainable was 70. A high score represents a higher dependency on masculinity to increase their self-worth. Further, the full scale indicated excellent reliability with Cronbach’s alpha of 0.91 in this sample.

#### Moderator

*Behavioral Emotion Regulation Questionnaire (BERQ)*: The BERQ^47^assesses the coping strategies of individuals as a response to stressful events with five-point Likert scale responses 1 (almost never) to 5 (almost always). The range of total scores on each sub-scale is 4 to 20. It is a 20-item questionnaire including five coping strategies: seeking distraction, withdrawing, actively approaching, seeking social support, and ignoring. The alpha coefficient reported by the original study for the scales mentioned above was 0.86, 0.93, 0.91, 0.91, and 0.89, respectively. In the present study, Cronbach’s alpha of the mentioned sub-scales is 0.80 for seeking distraction, 0.88 for withdrawal, 0.85 for actively approaching, 0.86 for seeking social support, and 0.89 for ignoring. We have categorized the coping strategies of behavioral emotional regulation as positive (seeking distraction, actively approaching and seeking social support) and negative (withdrawal and ignoring), as proposed in the original study^47^. The positive and negative strategies have good Cronbach alpha of 0.89 and 0.88, respectively, in the present sample.

### Data analysis

The data, which was collected through electronic mode and pen-and-paper, were coded, merged, and cleaned for duplications and missing values. The data was analyzed using SPSS 25 and Smart PLS 4. Path analyses, including moderation, were performed using the Process function of Smart PLS 4. The Smart PLS 4 was considered a suitable software for this data as it reduces the influence of skewness and improves the results’ robustness. We used bias-corrected accelerated (BCa) bootstrapping method^64^. The results are presented in 95% confidence intervals.

### Ethical concerns

We adhered to the following ethical guidelines: Written informed consent was acquired from all participants before the study procedure. All men were informed about their willingness to participate voluntarily and other participation rights, such as confidentiality of the data, anonymity of their identity, and the right to withdraw at any given time. The data was treated with anonymity and confidentiality, and no personally identifiable information was collected. The participants were given complete information about the research study. The contact details of the investigating researchers were made available through the form for any queries from the participants. We have acquired the necessary approval to conduct this study.

## Results

The study includes data from three developing nations, namely, India (*n* = 321), Oman (*n* = 214), and Ethiopia (*n* = 288), with a total sample size of 823. Table 1 presents the descriptive characteristics of the demographic variables across the three countries. The results suggest that 79.4% of the men had more than 15 years of education in India, with 12.1% and 56.3% of the men with 15 years and above educational years from Oman and Ethiopia, respectively. Regarding employment status, 86.9%, 36%, and 34% of individuals were employed in India, Oman, and Ethiopia, respectively. Further, considering marital status, 55.4%, 36.9%, and 41.0% of men were in a union from India, Oman and Ethiopia, respectively. Interestingly, 56.4% of participants from India, all the participants from Oman, and 69.5% from Ethiopia reported that the gender norms in the society influenced their behavior and decision-making in society.

**Table 1:** The descriptive characteristics of the sample across the three countries (*N* = 823).

Variables	India *n* (%)	Oman *n* (%)	Ethiopia *n* (%)
Sample	321 (39.1)	214 (26.0)	288(34.9)
Education (in years)			
Below 15 years	66(20.6)	188(87.9)	126(43.7)
15 years and above	255(79.4)	26(12.1)	162(56.3)
Employment status			
Student	42(13.1)	117(54.7)	160(55.6)
Employed	279(86.9)	77(36)	98(34)
Unemployed	0	20(9.3)	30(10.4)
Marital status			
Not in a union	143(44.6)	135(63.1)	170(59.0)
In a union	178(55.4)	79(36.9)	118(41.0)
Belief in the influence of gender norms			
Yes	181(56.4)	214(100)	200(69.5)
No	140(43.6)	0	88(30.5)

Table 2 presents the correlation between the continuous variables under the study. The mean age of the participants was 29 years, with a standard deviation of 7.79. Considering the mean of self-care (M = 73.93, SD = 15.64) and self-compassion (M = 38.12, SD = 6.49), it is evident that 49.9% and 56.3% of participants in the sample had reported poor involvement in self-care and had poor self-compassion. Further, 21.9% of the men in the sample had reported to have faced 4 or greater adverse childhood experiences. In addition, 50.7% of the men in the sample had a higher level of masculinity, considering the mean (41.99). Further, concerning the mean cutoff for the components of BER, 46.9% of men in the sample were involved in seeking distraction (M = 14.06, SD = 3.97). In comparison, 45.9% and 49.7% actively approached stressors or problems and sought social support during crisis, respectively, which are considered positive components of BER. Further, attributing to the negative form of handling a crisis or stressful events, 47.5% and 44.6% of the participants in the sample choose to withdraw (M = 13.22, SD = 4.63) and ignoring (M = 13.17, SD = 4.42) the stressor and problem, respectively.

The results of correlation analysis suggest that the higher levels of ACEs and masculinity were positively associated with self-care behavior (*r* =.13, *p* <.01; *r* =.21, *p* <.01, respectively). However, an increase in the level of ACEs reduced the likelihood of self-compassion (*r*=-.13, *p* <.01). The positive association between ACEs and self-care can be attributed to the sample characteristic as only minuscule participants had witnessed adverse experiences during their childhood (17.8%). Interestingly, all the five components of BER, namely seeking distraction (*r* =.44, *p* <.01), withdrawal (*r* =.19, *p* <.01), actively approaching (*r* =.38, *p* <.01), seeking support (*r* =.45, *p* <.01), and ignoring (*r* =.33, *p* <.01), were positively associated with self-care. It is essential to note that ACEs were positively associated with negative factors of BER, that is, coping through withdrawal and ignoring, and positive components of BER as seeking distraction and seeking support. In addition, a higher level of masculinity was positively associated with positive (seeking distraction, actively approaching and seeking support) and negative (withdrawal and ignoring) factors of BER. This positive association between masculinity and positive coping could be attributed to the nature of masculinity. In addition, this could be attributed to the differences in subjective masculinity features. Currently, with the rise in positive masculinity, this association between positive BER and masculinity requires substantiation in future works.

**Table 2:** The correlation between the continuous variables under study.

Variables	M (SD)	1	2	3	4	5	6	7	8	9
1. Self-care	73.93(15.64)	1								
2. Self-compassion	38.12(6.49)	0.12**	1							
3. ACEs	2.31(3.05)	0.13**	− 0.13**	1						
4. Masculinity	41.99 (14.87)	0.21**	− 0.05	0.31**	1					
5. Seeking Dis.	14.06 (3.97)	0.44**	0.18**	0.11**	0.07*	1				
6. Withdrawal	13.22 (4.63)	0.19**	− 0.33**	0.25**	0.18**	0.40**	1			
7. Actively App.	15.41 (3.53)	0.38**	0.17**	0.05	0.23**	0.56**	0.41**	1		
8. Seeking support	13.48 (4.47)	0.45**	− 0.04	0.09**	0.11*	0.56**	0.34**	0.49**	1	
9. Ignoring	13.17 (4.42)	0.33**	− 0.12**	0.16**	0.23**	0.47**	0.50**	0.44**	0.52**	1

Notes: Dis.- Distraction; App. Approaching; M – Mean; SD – Standard Deviation; ACEs (< 4) = 642; ACE’s (> = 4) = 181; *p <.05; **p <.01.

**Fig. 2:**
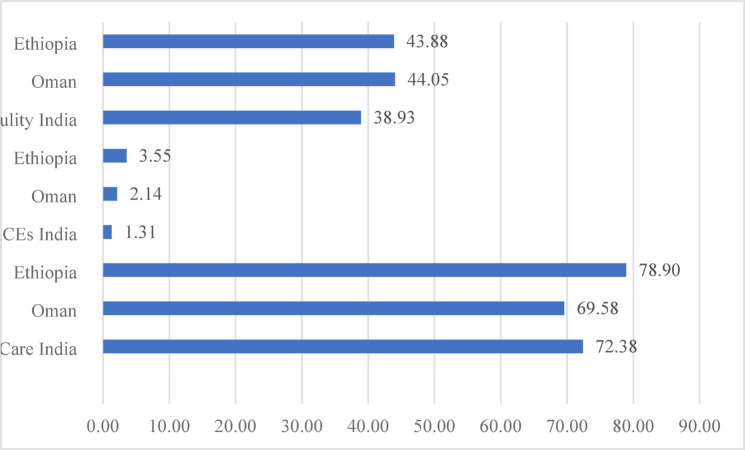
Mean difference between the three countries in terms of self-care, ACEs, and masculinity.

Table 3 summarises one-way ANOVA results for mean differences in ACEs, masculinity, and self-care across the three countries. The results point to the significant difference between India, Oman, and Ethiopia in terms of self-care (F (2,820) = 25.82, *p* <.001) ACEs (F (2,820) = 45.60, *p* <.001) and masculinity (F (2,820) = 11.41, *p* <.001). Figure 2 presents these results graphically, and it is evident that men from Ethiopia had higher ACEs than Oman and India. Further, men of Oman had a higher level of masculinity and lower involvement in self-care. In comparison, men in Ethiopia had a higher level of masculinity and good self-care involvement than men in India and Oman. Men in India indicated a lower level of masculinity than in the other two countries and higher involvement in self-care behavior than in Oman.

**Table 3:** Summary of one-way ANOVA results for self-care, aces, and masculinity among men of three countries.

Variables	Nationality	Mean	Mean difference	F (2, 820)	*p*
Self-care	India	72.38		25.82	< 0.001
	Oman	69.58	2.8		
	Ethiopia	78.90	−6.51		
ACEs	India	1.31		45.60	< 0.001
	Oman	2.14	− 0.82		
	Ethiopia	3.55	−2.23		
Masculinity	India	38.93		11.41	< 0.001
	Oman	44.05	−5.11		
	Ethiopia	43.88	−4.94		

**Fig. 3:**
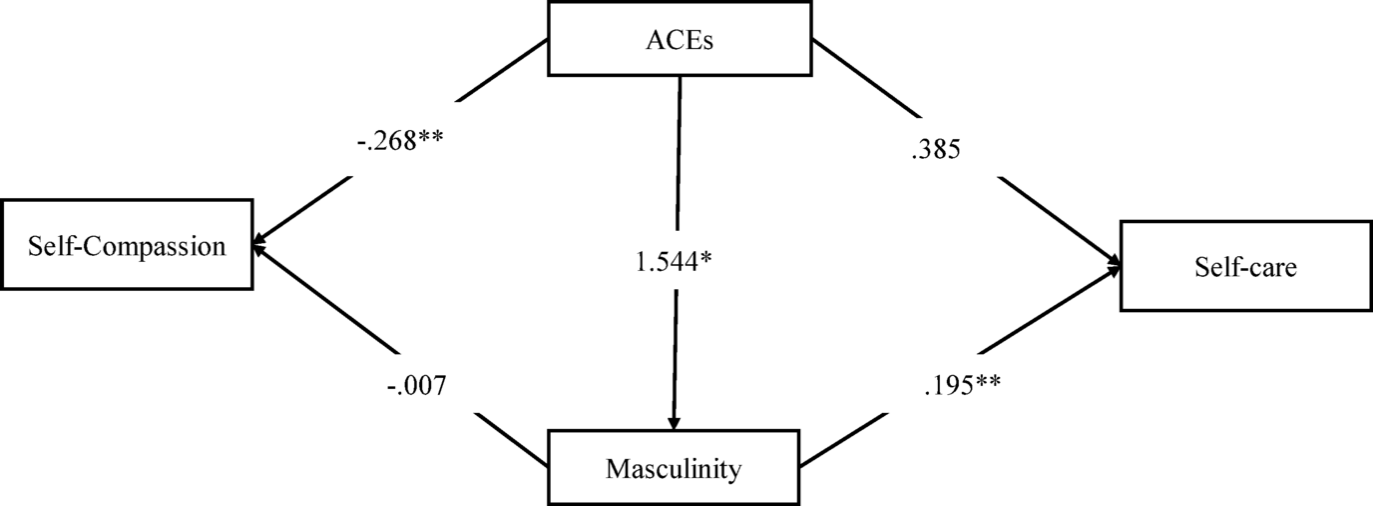
Path coefficients for self-care and self-compassion as outcomes. **p < 0.01, *p < 0.05.

Table 4 presents the summary of path analysis with self-care and self-compassion as the outcome. The results suggested a positive association between ACEs and masculinity, pointing at the increase in the likelihood of masculinity with an increase in ACEs (b = 1.544; 99% CI = 1.227–1.853) among the study individuals. In addition, an increase in ACEs reduced self-compassion among men in the sample (b=−0.268; 99% CI = − 0.375- − 0.157). Further, it is evident from the results that masculinity had a positive influence on men’s self-care, pointing at the increase in self-care with the increase in the levels of masculinity (b = 0.195; 99% CI = 0.097- 0.295). In addition, a significant indirect positive association existed between ACEs and self-care through masculinity (b = 0.302; 99% CI = 0.145- 0.482) (Refer to Fig. 3).

**Table 4:** Summary of path analysis results with self-care and self-compassion as outcomes.

Paths	B	95% CI		*p*
		**Lower**	**Upper**	
ACEs -> Masculinity	1.544	1.227	1.853	< 0.001
ACEs -> Self-care	0.385	− 0.067	0.800	0.085
ACEs -> Self-compassion	− 0.268	− 0.375	− 0.157	< 0.001
Masculinity -> Self-care	0.195	0.097	0.295	< 0.001
Masculinity -> Self-compassion	− 0.007	− 0.039	0.020	0.613
Total indirect effect				
ACEs -> Self-care	0.302	0.145	0.482	< 0.001
ACEs -> Self-compassion	− 0.011	− 0.059	0.031	0.617

Note: Bootstrapped with 1000; Model summary for self-care as an outcome: R^2^ = 0.049, t = 2.38, *p* <.05. Model summary for self-care as an outcome: R^2^ = 0.018, t = 2.52, *p* <.05.

**Table 5:** Summary of path analysis results with the negative and positive components of BER as moderators.

Paths	B	95% CI		*p*
		**Lower**	**Upper**	
Masculinity -> Self-care	− 0.644	− 0.988	− 0.281	< 0.001
Negative BER-> Self-care	−1.185	−2.280	− 0.125	0.031
Positive BER -> Self-care	1.416	0.109	2.783	0.037
Negative BER x masculinity -> Self-care	0.027	0.003	0.051	0.032
Positive BER x Masculinity -> Self-care	0.024	− 0.006	0.052	0.113

Note: Bootstrapped with 1000; Model summary for self-care as an outcome: R^2^ = 0.327, Adjusted R^2^ = 0.323, t = 10.59, *p* <.001.

**Fig. 4:**
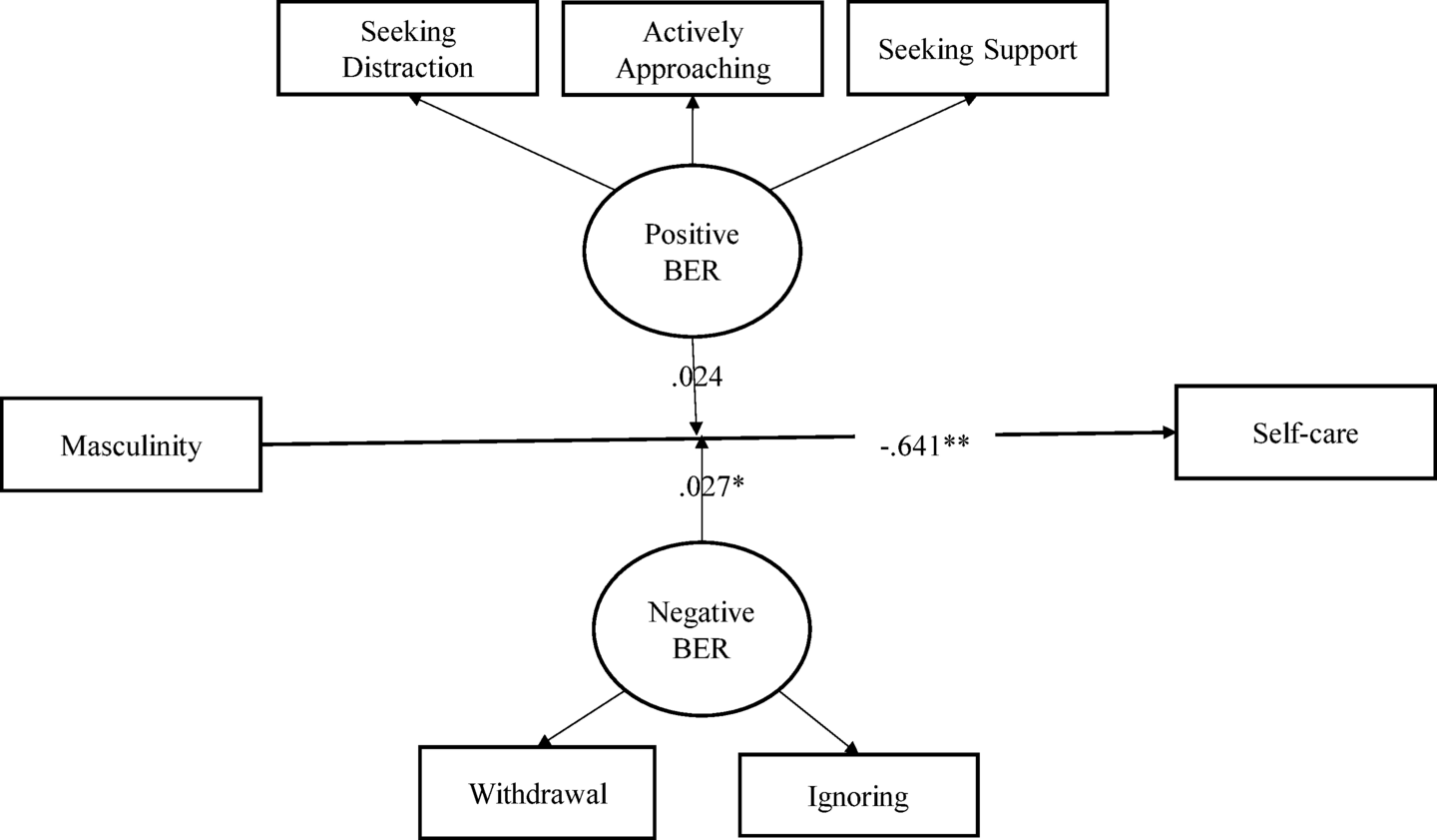
Moderation model with positive and negative components of BER as moderator. **p < 0.01, *p < 0.05.

Table 5 presents the path analysis results with negative and positive components of BER as moderators. It is evident from the results that the presence of emotional regulation as a moderator changed the direction between masculinity and self-care (b=−0.644; 99% CI = − 0.988- − 0.281). The results suggest that men coping through negative BER had a lower likelihood of involvement in self-care (b=−1.185; 95% CI= −2.280- − 0.125) while coping through positive BER increased the possibility of self-care (b = 1.416; 95% CI = 0.109- 2.783) among men. In addition, the components of negative BER acted as a moderator between masculinity and self-care (b = 0.027; 95% CI = 0.003–0.051), strengthening the negative association between masculinity and self-care (Refer to Fig. 4). The 32% of the variance in self-care was attributed to masculinity and positive and negative components of BER in this model.

## Discussion

In this study, we analyzed ACEs, masculinity contingency, and BER as they concern men’s self-compassion and involvement in self-care across three developing countries. Early childhood experiences, especially ACEs, are highly recognized in deciding the course of health and behavior outcomes throughout life. These outcomes differ among men and women^29,65^. Many studies show more adverse effects of ACEs for females compared to male counterparts^66^. On the other hand, this is also the case with traditional gender norms^67^. Complying with gender norms projects certain risks for poor maintenance of one’s mental health^68^. Although men possess the privilege of experiencing the overarching benefits of societal gender norms, it poses challenges to their behavior, well-being, and overall health^69^. Hence, we were interested in determining how ACEs and masculinity contingency are associated with two important health and well-being constructs^70^, self-compassion and self-care among men. In the latter part of the study, we present the role of positive and negative BER in how masculinity and self-care are associated.

Further, numerous studies have considered investigating self-care among patients with hypertension and diabetes and older adults with ill health^71–75^. In this regard, our study is unique in emphasizing the significance of engaging in self-care even among non-patient samples. Most of the samples’ below-average levels of self-compassion and engagement in self-care are concerning. In addition, the belief that restrictive gender norms influenced men’s behavior and decision-making in society was common among more than 80% of this study’s sample. This observation differed from those of Western countries, where contemporary beliefs about masculinity are shifting from traditional gender roles^76^.

The correlation between ACEs, masculinity, self-compassion, BER, and self-care was in line with the literature^77–79^. ACEs, masculinity, and self-care differed significantly across India, Oman, and Ethiopia. Furthermore, the findings from path analysis indicated that masculinity fully mediated the relationship between ACEs and self-care but not between ACEs and self-compassion. Men often experience threats to their masculinity by obeying or disobeying the social constructions of gender norms relating to masculinity or what it means to be a “man”^63,80^. The ideologies around masculinity among men vary, and ACEs constitute one of the developmental antecedents^78,81^. Similarly, the results of this study suggest that when men experience ACEs, the likelihood of masculinity increases. However, there is also an increase in self-care, contrastingly. In general, the earlier works have pointed at the increase in risky health behaviors with more conformity to traditional masculine norms^33,35^, which could possibly reduce self-care.

However, in line with present results, a study conducted among emerging adult men in the United States pointed at the positive role of masculine status (thriving for power and success) on mental health service utilization while toughness and anti-feminity aspects of masculine norm hindered it^39^. The nature of masculinity is evolving with the transition towards positive masculinity that could potentially promote self-care and well-being among men^41,82^. This transition is quite evident among college graduates in India as per a recent Pew research report on gender roles in Indian families^83^. In addition, the differences in results can also be attributed to the persisting dilemma among men and women in following traditional and modern norms, as the majority said responsibility should be shared in the household while they also supported traditional gender norms^83^. Similar patterns are expected in other two nations, though there exists no explicit work on these aspects to our knowledge.

Moreover, the moderation analysis results show that the involvement in negative BER changed the direction between masculinity and self-care, strengthening the negative association between the two variables. To our knowledge, earlier works have not directly studied these combinations of factors. Further, the negative relationship between masculinity and self-care was not mitigated by any of the three positive BER. However, in contrast, a previous study found that men who did not conform to masculine norms possessed high levels of self-care, moderated by a positive coping mechanism- positive reappraisal^84^. In addition, the results differ from health behavior change theory^85^, which suggests that positive coping mechanisms foster health-improving behaviors. Future studies should test this association to substantiate the results, and a deeper understanding of the subjective nature of masculinity through qualitative studies and its role in BER should be required.

The study results suggest interventions for reducing negative BER that could promote self-care among men. In addition, the self-care programs should be gender specific, focusing on BER components. This could be effectively done by promoting positive masculinity, training men in channelling emotions and enhancing involvement in positive coping techniques. It is pertinent to promote self-care among men through community, educational institutions or healthcare provisions to promote their well-being. In such programs, their notions about traditional masculine norms should be understood and mitigated, resulting in better emotional regulations. Also, it is important to consider ACEs, and psychosocial interventions should be initiated to mitigate the negative effects of ACEs in adulthood. In addition, it becomes essential to understand the evolving nature of masculinity in these countries as there exists the possibility of cultural transition with the dilemma of adopting the changing gender roles.

### Limitations and future directions

Besides the strengths of the present study, which includes samples from three different nations, the findings must be applied carefully, considering some of the important limitations. First, the study is cross-sectional in nature and does not provide a cause-effect explanation of the variables under study. Therefore, we suggest that future studies adopt a longitudinal design to understand the cause-effect relationship between the variables. Second, the study used self-reported measures. Although their reliability is well established, they may not be free of biases. Third, we did not consider the timing of occurrence and intensity of the ACEs, which may have been crucial in determining the negative effects on the respondents’ masculinity contingency. Future research can include these aspects of adverse childhood experiences while studying their association with other variables. Fourth, although we have employed probability sampling techniques to avoid selection bias, the sample might not represent the target population in all the countries. Hence, the study’s findings have limitations in terms of generalizability beyond the specific geographical contexts. Fifth, although the data was combined from India, Oman, and Ethiopia to test the hypothesized relationships, care must be taken while generalizing the results to a particular country due to the different cultural orientations and significant differences obtained in the study variables: ACEs, masculinity, and self-care among the samples of these countries. Therefore, there is scope for replicating the present study to confirm the plausibility of the findings across the three nations. In the future, qualitative studies can yield insights about the gender norms men are exposed to as children through their daily lives. In addition, future studies can explore the evolving nature of masculinity with a positive orientation that diverts from the traditional norms.

## Conclusion

The findings from the present study emphasize the association between ACEs, masculinity contingency, and important well-being-related constructs, namely, self-compassion and self-care among men in three countries. Further, there existed a positive association between ACEs and masculinity, contrastingly increasing self-care behavior. In addition, involvement in negative BER changed the direction between masculinity and self-care, strengthening the negative association between the two variables. Based on the evidence from this study, individuals must be taught healthy ways of deriving their self-worth rather than determining it from their masculine features. Our findings also have implications for educational institutions, which need to teach and normalize certain gender norms and not burden male students with specific ways of being perceived as a “macho” or a “man.” This practice should continue to exist even outside the institutional premises. The parents must be educated about gender norms and their negative impacts on health and other harmful behaviors in adulthood. More opportunities for mental health services should be created so that men can seek support and actively approach any problem throughout their life course.

## Data Availability

The data used for this study is available on request from the corresponding author.

## References

[1] World Health Organization (WHO). Tenth Global Conference on Health Promotion. (2021). https://www.who.int/teams/health-promotion/enhanced-wellbeing/tenth-global-conference-on-health-promotion

[2] United Nations— SDG Indicators. (2023). https://unstats.un.org/sdgs/report/.

[3] Pappachan, M. J. Increasing prevalence of lifestyle diseases: high time for action. *Indian J. Med. Res.* **134**, 143–145 (2011). 21911964 PMC3181012

[4] Wang, Y. & Wang, J. Modelling and prediction of global non-communicable diseases. *BMC Public. Health*. **20**, 822 (2020). 32487173 10.1186/s12889-020-08890-4 PMC7268487

[5] Lancet Global, regional, and National burden of 12 mental disorders in 204 countries and territories, 1990–2019: a systematic analysis for the global burden of disease study 2019. *Lancet Psychiatry*. **9**, 137–150 (2022). 35026139 10.1016/S2215-0366(21)00395-3 PMC8776563

[6] Perera, N. & Agboola, S. Are formal self-care interventions for healthy people effective? A systematic review of the evidence. *BMJ Glob Health*. **4**, e001415 (2019). 31799010 10.1136/bmjgh-2019-001415 PMC6861059

[7] Martínez, N., Connelly, C. D., Pérez, A. & Calero, P. Self-care: A concept analysis. *Int. J. Nurs. Sci.* **8**, 418–425 (2021). 34631992 10.1016/j.ijnss.2021.08.007 PMC8488814

[8] Torres-Soto, N. Y., Corral-Verdugo, V. & Corral-Frías, N. S. The relationship between self-care, positive family environment, and human well-being. *Well-being Space Soc.* **3**, 100076 (2022).

[9] Riegel, B. et al. Self-Care for the prevention and management of cardiovascular disease and stroke. *J. Am. Heart Assoc.* **6**, e006997 (2017). 28860232 10.1161/JAHA.117.006997 PMC5634314

[10] World Health Organization (WHO). Self-care for health and well-being. (2024). https://www.who.int/news-room/fact-sheets/detail/self-care-health-interventions

[11] Homan, K. J. & Sirois, F. M. Self-compassion and physical health: exploring the roles of perceived stress and health-promoting behaviors. *Health Psychol. Open.* **4**, 2055102917729542 (2017). 29379620 10.1177/2055102917729542 PMC5779931

[12] Biber, D. D. & Ellis, R. The effect of self-compassion on the self-regulation of health behaviors: A systematic review. *J. Health Psychol.* **24**, 2060–2071 (2019). 28810473 10.1177/1359105317713361

[13] Sahdra, B. K. et al. The compassion balance: Understanding the interrelation of Self- and Other-Compassion for optimal Well-being. *Mindfulness* **14**, 1997–2013 (2023).

[14] Cowand, A., Amarsaikhan, U., Ricks, R. F., Cash, E. D. & Sephton, S. E. Self-Compassion is associated with improved Well-Being and healthier cortisol profiles in undergraduate students. *Mindfulness* **15**, 1831–1845 (2024).

[15] Lee, E. E. et al. Compassion toward others and self-compassion predict mental and physical well-being: a 5-year longitudinal study of 1090 community-dwelling adults across the lifespan. *Transl Psychiatry*. **11**, 1–9 (2021). 34282145 10.1038/s41398-021-01491-8 PMC8287292

[16] Garbutt, K., Rennoldson, M. & Gregson, M. Shame and self-Compassion connect childhood experience of adversity with harm inflicted on the self and others. *J. Interpers. Violence*. **38**, 7193–7214 (2022). 36541192 10.1177/08862605221141866 PMC10170577

[17] Thomas, V. Does Self-care Moderate the Association between Adverse Childhood Experiences, Trauma Symptoms, and Parental Reflective Functioning? (2024).

[18] Crouch, E., Probst, J. C., Radcliff, E., Bennett, K. J. & McKinney, S. H. Prevalence of adverse childhood experiences (ACEs) among US children. *Child. Abuse Negl.* **92**, 209–218 (2019). 31003066 10.1016/j.chiabu.2019.04.010

[19] Tzouvara, V. et al. Adverse childhood experiences, mental health, and social functioning: A scoping review of the literature. *Child. Abuse Negl.* **139**, 106092 (2023). 36907117 10.1016/j.chiabu.2023.106092

[20] Ramiro, L. S., Madrid, B. J. & Brown, D. W. Adverse childhood experiences (ACE) and health-risk behaviors among adults in a developing country setting. *Child. Abuse Negl.* **34**, 842–855 (2010). 20888640 10.1016/j.chiabu.2010.02.012

[21] Maurya, C. & Maurya, P. Adverse childhood experiences and health risk behaviours among adolescents and young adults: evidence from India. *BMC Public. Health*. **23**, 536 (2023). 36944936 10.1186/s12889-023-15416-1 PMC10031876

[22] Testa, A. et al. Adverse childhood experiences and unhealthy dietary behaviours in adulthood. *Public. Health Nutr.* **27**, e40 (2024). 38234114 10.1017/S1368980024000144 PMC10882537

[23] Salokangas, R. K. R., From, T., Luutonen, S. & Hietala, J. Adverse childhood experiences leads to perceived negative attitude of others and the effect of adverse childhood experiences on depression in adulthood is mediated via negative attitude of others. *Eur. Psychiatry*. **54**, 27–34 (2018). 30041073 10.1016/j.eurpsy.2018.06.011

[24] Merrick, M. T. et al. Unpacking the impact of adverse childhood experiences on adult mental health. *Child. Abuse Negl.* **69**, 10–19 (2017). 28419887 10.1016/j.chiabu.2017.03.016 PMC6007802

[25] Wakuta, M. et al. Adverse childhood experiences: impacts on adult mental health and social withdrawal. *Front. Public. Health*. **11**, 1277766 (2023). 37954050 10.3389/fpubh.2023.1277766 PMC10639139

[26] Daníelsdóttir, H. B. et al. Adverse childhood experiences and adult mental health outcomes. *JAMA Psychiatry*. **81**, 586–594 (2024). 38446452 10.1001/jamapsychiatry.2024.0039 PMC10918580

[27] Daines, C. L., Hansen, D., Novilla, M. L. B. & Crandall, A. Effects of positive and negative childhood experiences on adult family health. *BMC Public. Health*. **21**, 651 (2021). 33820532 10.1186/s12889-021-10732-w PMC8022401

[28] Mlouki, I. et al. Gender differences in adverse childhood experiences, resilience and internet addiction among Tunisian students: exploring the mediation effect. *PLOS Glob Public. Health*. **4**, e0002556 (2024). 38236830 10.1371/journal.pgph.0002556 PMC10795992

[29] Jones, M. S., Pierce, H. & Shafer, K. Gender differences in early adverse childhood experiences and youth psychological distress. *J. Crim Justice*. **83**, 101925 (2022).

[30] Fleming, P. J. & Agnew-Brune, C. Current trends in the study of gender norms and health behaviors. *Curr. Opin. Psychol.* **5**, 72–77 (2015). 26075291 10.1016/j.copsyc.2015.05.001 PMC4461071

[31] Rice, S. et al. Gender norms and the mental health of boys and young men. *Lancet Public. Health*. **6**, e541–e542 (2021). 34332667 10.1016/S2468-2667(21)00138-9

[32] Saltonstall, R. Healthy bodies, social bodies: Men’s and women’s concepts and practices of health in everyday life. *Soc. Sci. Med.* **1982 36**, 7–14 (1993). 10.1016/0277-9536(93)90300-s 8424186

[33] Merdassa, A. B. Traditional masculinity, peer pressure, and sensation seeking as correlates of risky behaviours. *Int. J. Adolesc. Youth*. **29**, 2298087 (2024).

[34] Bel-Latour, L. & Granié, M. A. The influence of the perceived masculinity of an occupation on risk behavior: the case of firefighters. *Saf. Sci.* **150**, 105702 (2022).

[35] Pomerantz, A. L., Foster, S. & Bell, K. Invincible honor: masculine honor, perceived invulnerability, and risky decision-making. *Curr. Psychol. N B Nj*. **1–9**10.1007/s12144-023-04722-x (2023). 10.1007/s12144-023-04722-x PMC10170145 37359611

[36] Milner, A., Shields, M. & King, T. The influence of masculine norms and mental health on health literacy among men: evidence from the ten to men study. *Am. J. Mens Health* **13**, (2019). 10.1177/1557988319873532 PMC6728685 31690213

[37] Ezeugwu, C. R. & Ojedokun, O. Masculine norms and mental health of African men: what can psychology do? *Heliyon* 6, e05650 (2020). 10.1016/j.heliyon.2020.e05650 PMC7734219 33336092

[38] Herreen, D., Rice, S., Currier, D., Schlichthorst, M. & Zajac, I. Associations between conformity to masculine norms and depression: age effects from a population study of Australian men. *BMC Psychol.* **9**, 32 (2021). 33608063 10.1186/s40359-021-00533-6 PMC7893732

[39] Sileo, K. M. & Kershaw, T. S. Dimensions of masculine norms, depression, and mental health service utilization: results from a prospective cohort study among emerging adult men in the united States. *Am. J. Mens Health*. **14**, 1557988320906980 (2020). 32079448 10.1177/1557988320906980 PMC7036518

[40] Fidolini, V. Eating like a man. Food, masculinities and self-care behavior. *Food Cult. Soc.* **25**, 254–267 (2022).

[41] Wilson, M. J. Cultivating positive masculinity is mental health promotion for boys and men. *Health Promot Int.* **37**, daac121 (2022). 36047638 10.1093/heapro/daac121

[42] Iacoviello, V., Valsecchi, G., Berent, J., Borinca, I. & Falomir-Pichastor, J. M. Is traditional masculinity still valued?? Men’s perceptions of how different reference groups valued? traditional masculinity norms. *J. Men’s Stud.* **30**, 7–27 (2022).

[43] Jakupcak, M., Salters, K., Gratz, K. L. & Roemer, L. Masculinity and emotionality: an investigation of Men’s primary and secondary emotional responding. *Sex. Roles*. **49**, 111–120 (2003).

[44] Logoz, F. et al. How do traditional masculinity ideologies and emotional competence relate to aggression and physical domestic violence in cisgender men? *Front. Psychol.* **14**, (2023). 10.3389/fpsyg.2023.1100114 PMC10043379 36998370

[45] Flynn, J. J., Hollenstein, T. & Mackey, A. The effect of suppressing and not accepting emotions on depressive symptoms: is suppression different for men and women? *Personal Individ Differ.* **49**, 582–586 (2010).

[46] Kozubal, M., Szuster, A. & Wielgopolan, A. Emotional regulation strategies in daily life: the intensity of emotions and regulation choice. *Front. Psychol.* **14**, 1218694 (2023). 37645071 10.3389/fpsyg.2023.1218694 PMC10460911

[47] Kraaij, V. & Garnefski, N. The behavioral emotion regulation questionnaire: development, psychometric properties and relationships with emotional problems and the cognitive emotion regulation questionnaire. *Personal Individ Differ.* **137**, 56–61 (2019).

[48] Le, T. T. & Jin, R. Vortex of regret: how positive and negative coping strategies correlate with feelings of guilt. *Acta Psychol. (Amst)*. **247**, 104320 (2024). 38762956 10.1016/j.actpsy.2024.104320

[49] Bloom, S. L. Trauma theory. In *Humanizing Mental Health Care in Australia: A Guide To trauma-informed Approaches* 3–30 (Routledge/Taylor & Francis Group, 2019). 10.4324/9780429021923-1.

[50] Treatment (US), C. for S. A. Understanding the impact of trauma. in Trauma-Informed Care in Behavioral Health Services (Substance Abuse and Mental Health Services Administration (US), (2014). 24901203

[51] Baker, P. Men’s health: time for a new approach. *Phys. Ther. Rev.* (2018).

[52] Vandello, J. A., Wilkerson, M., Bosson, J. K. & Wiernik, B. M. Kosakowska-Berezecka, N. Precarious manhood and Men’s physical health around the world. *Psychol. Men Masculinities*. 10.1037/men0000407 (2022).

[53] Al Azri, Z., Al-abri, K., Sawafi, A. & Jaju, A. Al Qadire, M. Adverse childhood experiences and risky behaviors in Oman: A cross-sectional study. *Prev. Med. Rep.* **44**, 102809 (2024). 39071240 10.1016/j.pmedr.2024.102809 PMC11277357

[54] Sunitha, S. & Gururaj, G. Health behaviours & problems among young people in India: cause for concern & call for action. *Indian J. Med. Res.* **140**, 185–208 (2014). 25297351 PMC4216492

[55] Maurya, P., Sinha, D. & Chattopadhyay, A. Non-Communicable Disease among Men in India: How far Occupation and Health Behaviour Matter? (2020).

[56] Al-Mawali, A. et al. Prevalence of risk factors of non-communicable diseases in the Sultanate of Oman: STEPS survey 2017. *PLoS ONE*. **16**, e0259239 (2021). 34710161 10.1371/journal.pone.0259239 PMC8553065

[57] Gelaw, Y. A. et al. Socio-demographic correlates of unhealthy lifestyle in Ethiopia: a secondary analysis of a National survey. *BMC Public. Health*. **23**, 1528 (2023). 37568091 10.1186/s12889-023-16436-7 PMC10416504

[58] Baker, P., Leon, N., Colvin, C. J. & Griffith, D. M. Health policies must consider gender, including men. *Lancet Glob Health*. **11**, e1847–e1848 (2023). 37973331 10.1016/S2214-109X(23)00428-X

[59] Luciani, M. et al. Measuring self-care in the general adult population: development and psychometric testing of the Self-Care inventory. *BMC Public. Health*. **22**, 1–10 (2022). 35346104 10.1186/s12889-022-12913-7 PMC8960109

[60] Raes, F., Pommier, E., Neff, K. D. & Van Gucht, D. Construction and factorial validation of a short form of the Self-Compassion scale. *Clin. Psychol. Psychother.* **18**, 250–255 (2011). 21584907 10.1002/cpp.702

[61] Felitti, V. J. et al. Relationship of childhood abuse and household dysfunction to many of the leading causes of death in adults: the adverse childhood experiences (ACE) study. *Am. J. Prev. Med.* **14**, 245–258 (1998). 9635069 10.1016/s0749-3797(98)00017-8

[62] Murphy, A. et al. Adverse childhood experiences (ACEs) questionnaire and adult attachment interview (AAI): implications for parent child relationships. *Child. Abuse Negl.* **38**, 224–233 (2014). 24670331 10.1016/j.chiabu.2013.09.004

[63] Burkley, M., Wong, Y. J. & Bell, A. C. The masculinity contingency scale (MCS): scale development and psychometric properties. *Psychol. Men Masculinity*. **17**, 113–125 (2015).

[64] Hair, J. F., Risher, J. J., Sarstedt, M. & Ringle, C. M. When to use and how to report the results of PLS-SEM. *Eur. Bus. Rev.* **31**, 2–24 (2019).

[65] Leban, L. & Gibson, C. L. The role of gender in the relationship between adverse childhood experiences and delinquency and substance use in adolescence. *J. Crim Justice*. **66**, 101637 (2020).

[66] Whitaker, R. C. et al. The interaction of adverse childhood experiences and gender as risk factors for depression and anxiety disorders in US adults: a cross-sectional study. *BMC Public Health* 21, (2021). (2078). 10.1186/s12889-021-12058-z PMC8590371 34772386

[67] UNICEF. Gender equality | UNICEF. https://www.unicef.org/gender-equality

[68] Rice, S. M., Purcell, R. & McGorry, P. D. Adolescent and young adult male mental health: transforming system failures into proactive models of engagement. *J. Adolesc. Health Off Publ Soc. Adolesc. Med.* **62**, S9–S17 (2018). 10.1016/j.jadohealth.2017.07.024 29455724

[69] Heise, L. et al. Gender inequality and restrictive gender norms: framing the challenges to health. *Lancet Lond. Engl.* **393**, 2440–2454 (2019). 10.1016/S0140-6736(19)30652-X 31155275

[70] WHO. Self-care for health and well-being. (2024). https://www.who.int/news-room/fact-sheets/detail/self-care-health-interventions

[71] D’Souza, M. S. et al. Self-efficacy and self-care behaviours among adults with type 2 diabetes. *Appl. Nurs. Res.* **36**, 25–32 (2017). 28720235 10.1016/j.apnr.2017.05.004

[72] Dasappa, H., Prasad, S., Sirisha, M., Ratna Prasanna, S. V. N. & Naik, S. Prevalence of self-care practices and assessment of their sociodemographic risk factors among diabetes in the urban slums of Bengaluru. *J. Fam Med. Prim. Care*. **6**, 218–221 (2017). 10.4103/2249-4863.220037 PMC5749060 29302521

[73] Gangopadhyay, J. Ageing and Self-Care in India: examining the role of the market in determining a new course of growing old among middle class older adults in urban India. *Ageing Int.* **47**, 801–815 (2022).

[74] Emire, M. S. et al. Self-care practice and its associated factors among diabetic patients attending public hospitals in gurage zone Southwest, Ethiopia. *PloS One*. **17**, e0271680 (2022). 36155496 10.1371/journal.pone.0271680 PMC9512188

[75] Dagnew, B., Debalkie Demissie, G. & Abebaw Angaw, D. Systematic Review and Meta-Analysis of Good Self-Care Practice among People Living with Type 2 Diabetes Mellitus in Ethiopia: A National Call to Bolster Lifestyle Changes. *Evid. Based Complement. Alternat. Med.* 8896896 (2021). (2021). 10.1155/2021/8896896 PMC7920708 33688368

[76] Valsecchi, G., Iacoviello, V., Berent, J., Borinca, I. & Falomir-Pichastor, J. M. Men’s gender norms and gender-Hierarchy-Legitimizing ideologies: the effect of priming traditional masculinity versus a feminization of Men’s norms. *Gend. Issues*. **40**, 145–167 (2023). 10.1007/s12147-022-09308-8 PMC10689301 38044965

[77] Jay Miller, J., Lee, J., Niu, C., Grise-Owens, E. & Bode, M. Self-Compassion as a predictor of Self-Care: A study of social work clinicians. *Clin. Soc. Work J.* **47**, 321–331 (2019).

[78] Curtis, M. G., Oshri, A., Bryant, C. M., Bermudez, M. & Kogan, S. M. Contextual adversity and rural black Men’s masculinity ideology during emerging adulthood. *Psychol. Men Masculinity*. **22**, 217–226 (2021). 10.1037/men0000319 PMC8318358 34335107

[79] Garbutt, K., Rennoldson, M. & Gregson, M. Shame and self-Compassion connect childhood experience of adversity with harm inflicted on the self and others. *J. Interpers. Violence*. **38**, 7193–7214 (2023). 36541192 10.1177/08862605221141866 PMC10170577

[80] Magovcevic, M. & Addis, M. E. The masculine depression scale: development and psychometric evaluation. *Psychol. Men Masculinity*. **9**, 117 (2008).

[81] Zhang, J. & Zheng, L. Adverse childhood experiences predict preference for male facial masculinity in gay men in China. *Evol. Psychol. Sci.* **8**, 254–261 (2022).

[82] Sloan, C., Conner, M. & Gough, B. How does masculinity impact on health? A quantitative study of masculinity and health behavior in a sample of UK men and women. *Psychol. Men Masculinity*. **16**, 206–217 (2015).

[83] Corichi, J. E., Sahgal, N. & Salazar, A. M. Kelsey Jo Starr and Manolo. 3. Gender roles in the family. *Pew Res. Cent.* (2022). https://www.pewresearch.org/religion/2022/03/02/gender-roles-in-the-family/

[84] Davis, B., Honomichl, R. & Sullivan, A. B. Effects of conformity to masculine norms and coping on health behaviors in men with multiple sclerosis. *Int. J. MS Care*. **24**, 162–168 (2022). 35875456 10.7224/1537-2073.2020-116 PMC9296053

[85] Ryan, P. Integrated theory of health behavior change: background and intervention development. *Clin. Nurse Spec.* **23**, 161 (2009). 19395894 10.1097/NUR.0b013e3181a42373 PMC2778019

